# Predicting Difficult Tracheal Intubation Using Multi-Angle Photographic Analysis with Convolutional Neural Networks and EfficientNet

**DOI:** 10.3390/diagnostics16071042

**Published:** 2026-03-30

**Authors:** Erdinç Koca, Sevgi Kutlusoy, Mehmet Bilal Er, Tarkan Koca

**Affiliations:** 1 Anesthesiology and Reanimation Department, Malatya Inonu University, 44100 Malatya, Turkey; 2 Anesthesiology and Reanimation Department, Balıkesir Education and Training Hospital, 10000 Balıkesir, Turkey; 3 Department of Software Engineering, Harran University, 63000 Şanlıurfa, Turkey; bilal.er@harran.edu.tr; 4 Department of Machine Engineering, Inonu University, 44100 Malatya, Turkey

**Keywords:** intubation, photographic analysis, deep learning

## Abstract

**Background:** Difficult intubation is an important clinical problem faced by anesthesiologists and is one of the most important causes of anesthesia-related morbidity. According to various sources, the frequency of encountering a difficult airway is stated as 1–4%. **Aim:** We thought that difficult tracheal intubation could be predicted by photographic analysis using artificial intelligence. **Methods:** Sixteen photographs were taken in the preoperative period in the sitting and lying positions anteriorly, laterally, with the mouth open, with the mouth closed, with the neck straight, and with the neck extended. Intubations performed without intervention for the first time were considered easy. Intubations with external tracheal intervention and with more than one attempt were evaluated as medium. Intubations requiring more than three attempts; intubation with stylets, fiberoptic bronchoscopes, or video laryngoscopes; or cases in which patients could not be intubated and provided airway with a laryngeal mask were considered difficult. **Results:** In our study, the CNN (convolutional neural network) model performed well overall, with the best results generally obtained using batch sizes of 32 and 128 and learning rates ranging from 0.1 to 0.001. **Conclusions:** The prominent aspects of our study are that it can be conducted with an easily accessible mobile phone, can be performed at the bedside, and is successful in predicting difficult intubation. The sensitivity of methods currently used to assess difficult airways is generally low, and the likelihood of clinicians successfully identifying this condition using available information varies widely; thus far, there is no gold standard for prediction. We believe that our study will bring a different perspective to estimating the difficulty of intubation, which occupies a very important place in anesthesia practice.

## 1. Introduction

Endotracheal intubation is performed in the operating room to provide patients with safe upper airway patency and ensure maintenance of anesthesia. In intensive care and emergency units, it is performed to maintain respiration in patients whose breathing becomes superficial or whose breathing stops [1,2]. However, depending on the patient’s anatomical features, it is not always possible to perform endotracheal intubation successfully [1,3]. Although this situation is called difficult intubation, it has no universally accepted definition to date. Its definitions vary widely and include the need to change equipment or the physician performing intubation, more than two or three intubation attempts, intubation lasting longer than 10 min, and failed intubation [4].

Another definition, difficult laryngoscopy, refers to the inability to visualize parts of the vocal cords after several conventional laryngoscopy attempts by a trained anesthesiologist [5]. The American Society of Anesthesiologists (ASA) defines difficult airway management as follows: “The anesthesiologist has difficulty ventilating the upper airway through a face mask, has difficulty intubating the trachea, or both” [5]. Careful preoperative airway assessment is a top priority for anesthesiologists to improve the understanding, prevention, and management of airway-related complications [6]. However, there are some patients in whom even a skilled anesthesiologist experiences challenges predicting intubation difficulty [7]. Among the methods currently used to assess difficult airways are the upper lip bite test and the modified LEMON (an acronym for the assessment of the airway’s appearance, identification of any dental issues, evaluation of Mallampati classification, assessment of airway obstruction, and examination of neck mobility) criterion, but they have poor to medium discriminatory power when used alone or even in combination [8,9].

Other commonly used bedside airway examinations are the modified Mallampati test and thyromental distance measurement, which both have a sensitivity of 30% to 60% and a specificity of 60% to 80%, with low positive predictive values [10]. Intubation difficulty is observed at a rate of 1–13% and severe intubation difficulty at a rate of 2–3% [1]. This difficulty may cause severe brain damage and even death due to an inadequate oxygen supply [11,12]. Therefore, the accurate prediction of difficult intubation can significantly improve patient safety by enabling clinicians to take appropriate preparatory measures before the induction of anesthesia [13]. However, the sensitivity of methods currently used to assess difficult airways is generally low, and the likelihood of clinicians successfully identifying this condition using available information varies widely; thus far, there is no gold standard for prediction [14]. Equipment used in difficult intubation includes fiberoptic bronchoscopes, rigid laryngoscopes (Shikaani, Bullard, and Wu scopes), video laryngoscopes (Mac videoscope, Glidescope, and Airtraq), and intubation stylets. Cricothyrotomy and emergency tracheotomy can also be used in cases that cannot be intubated [15].

Inadequacy or failure of airway management in patients receiving general anesthesia is responsible for 30–40% of anesthesia-related deaths. An analysis of closed insurance cases against anesthesiologists (ASA closed claims) showed that 17% were related to difficult/impossible intubation without documented preoperative airway assessments [16]. In a study in Turkey, the rate of difficult intubation was reported to be 4.8%. In the same study, the best results were obtained when mouth opening or Mallampati was used alone, and it was emphasized that the combined use of tests did not provide a benefit in predicting difficult intubation [17].

The medical field is ripe for artificial intelligence (AI) applications that can learn over time to predict optimal treatments and minimize side effects [18]. The AI is currently being used in many areas of medicine to create programs that can perform clinical diagnostic procedures and provide treatment recommendations [19]. However, only a limited number of studies have focused on the prediction of difficult intubation using AI [20,21,22]. In our study, we aimed to predict difficult intubation (while taking the necessary precautions) using AI-assisted bedside photographs taken with a cellphone.

The main contributions of this study can be summarized as follows:

- A deep learning-based framework for predicting tracheal intubation difficulty using smartphone-acquired bedside photographs is proposed.
- Unlike many previous studies that focus on binary classification, this study introduces a three-class classification framework (easy, medium, difficult), enabling a more detailed evaluation of airway difficulty.
- Two deep learning architectures, CNN and EfficientNet, are implemented and comparatively evaluated for predicting intubation difficulty from multi-angle facial and neck photographs.
- A multi-angle photographic dataset consisting of 16 images per patient is utilized, allowing the models to capture richer anatomical information related to airway assessment.
- The effects of different batch sizes and learning rates are systematically analyzed to evaluate the robustness and stability of the proposed models.

The remainder of this paper is organized as follows. Section 2 describes the materials and methods used in this study, including the dataset, image acquisition process, preprocessing steps, and the deep learning architectures (CNN and EfficientNet) employed for the classification of intubation difficulty. Section 3 presents the experimental results and performance evaluation of the proposed models using metrics such as accuracy, precision, sensitivity, and F1-score. Section 4 discusses the results obtained and compares them with findings reported in previous studies in the literature. Finally, Section 5 concludes the study and highlights the limitations of the proposed approach as well as potential directions for future research.

## 2. Materials and Methods

**Figure 1:**
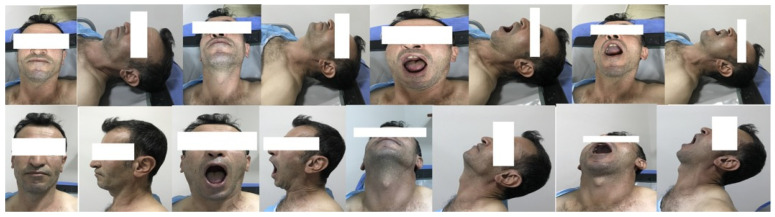
Eight patterns were captured in each of the supine and sitting positions for a total of 16 patterns.

The study commenced after approval from the Malatya Turgut Ozal University Clinical Research Ethics Committee (approval no. 2022/07) was obtained. A total of 109 patients who underwent general anesthesia at Malatya Training and Research Hospital were included in the study. Patients were excluded if they were under 18 years of age, had head and neck tumors, underwent emergency surgeries, had neurological deficits, cervical joint restriction, maxillofacial trauma, cervical vertebra trauma, laryngeal injury, or congenital malformations, were pregnant, or did not provide consent. After written informed consent was obtained from the patients, 16 photographs were taken in the preoperative period in the sitting and lying positions anteriorly, laterally, with the mouth open, with the mouth closed, with the neck straight, and with the neck extended. All images were taken with a smartphone (iPhone 7) (Figure 1). After the induction of anesthesia, the patients were positioned on the operating table and intubated using a Macintosh blade size 3–4 by an anesthesiologist. As shown in Table 1, the dataset contains patients categorized into three classes representing different levels of intubation difficulty.

**Figure 2:**
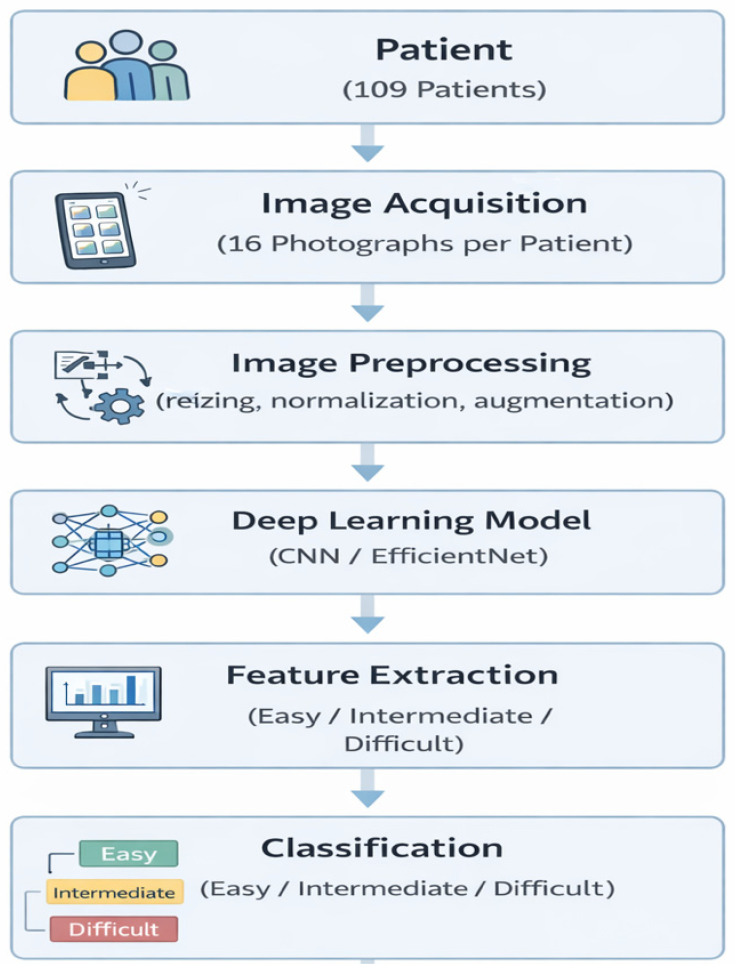
Overview of the proposed deep learning pipeline for predicting intubation difficulty.

Intubations performed without intervention for the first time were considered easy. Intubations with external tracheal intervention and with more than one attempt were evaluated as medium. Intubations requiring more than three attempts; intubation with stylets, fiberoptic bronchoscopes, or video laryngoscopes; or cases in which patients could not be intubated and provided airway with a laryngeal mask were considered difficult. Patients were screened for intubation difficulty using the photographs taken. In this study, the 16 photographs obtained from each patient were treated as independent input samples during model training. The overall data processing workflow used in this study is illustrated in Figure 2.

In our study, patients’ Mallampati scores and thyromental distances were recorded. Mallampati scores were found to be 1 and 2 in the easy group, 1, 2, and 3 in the moderate group, and 2 and 3 in the difficult group. Thyromental distances were found to be 5.1 cm to 11.6 cm in the easy group, 5.4 cm to 10.6 cm in the medium group, and 4.9 cm to 9.1 cm in the difficult group.

A feature fusion strategy combining multiple views into a single feature vector was not applied, and no ensemble of separate sub-networks was used. Instead, a single deep learning architecture (CNN or EfficientNet) was trained to classify the images directly into three intubation difficulty categories (easy, medium, and difficult). Each photograph captured from different anatomical perspectives contributes to the learning process by providing complementary visual information related to airway anatomy. This approach allows the deep learning models to learn discriminative features directly from individual images while benefiting from the diversity of multiple viewpoints. For each patient included in the study, 16 photographs were captured from different anatomical perspectives during the preoperative evaluation. These photographs included anterior and lateral views taken in both sitting and supine positions, with variations such as mouth open, mouth closed, neck in a neutral position, and neck extended. These multiple views were designed to capture different anatomical characteristics relevant to airway assessment. During model training, the images were treated as individual input samples, while the corresponding label for each image was determined according to the clinically observed intubation difficulty level of the patient (easy, medium, or difficult). All images were first resized to a fixed resolution and normalized before being fed into the deep learning models. To improve model robustness and reduce overfitting, data augmentation techniques such as horizontal flipping, rotation, and brightness adjustments were applied. The dataset was divided into training, validation, and test subsets, enabling reliable performance evaluation of the models. The CNN and EfficientNet architectures were trained using the Adam optimizer with categorical cross-entropy loss, and training was conducted for approximately 20–50 epochs depending on the convergence behavior of the models.

In this study, two deep learning (DL)-based models were used to classify the cases into easy, medium, and difficult: a CNN and EfficientNet. Both models are used to learn and classify image features, but EfficientNet has been proven to be more efficient and powerful, as it can achieve high classification accuracy with fewer parameters [23,24]. The dataset consisted of easy, medium, and difficult intubation images, each labeled with tags representing different levels of difficulty. These images were preprocessed for better learning of the model. Preprocessing included steps such as resizing the images, applying data augmentation techniques, and normalizing pixel values.

The CNN model is widely used in the field of DL, and in this study, the model was composed of convolutional layers to learn basic image features. Convolutional layers extract basic features, such as edges and corners, from an image and make the information compact using max pooling layers [25]. Classification is then performed for the three classes (easy, medium, and difficult) using fully connected layers and an output layer.

EfficientNet is a powerful model known for its high accuracy and need for fewer parameters. It optimizes depth, width, and resolution parameters to enable fast and efficient image classification. Categorical cross-entropy loss and the Adam optimizer were used to train the model in this study. The training process was optimized in the range of about 20–50 epochs, terminating at a point in which accuracy increased and the model stabilized. Model success was evaluated with metrics, such as accuracy, sensitivity, precision, and F1-score. The performance of the trained models was then compared to determine the differences between CNN and EfficientNet. After the classification accuracy and overall performance of both models were identified, the results were presented to the anesthesiologists to help them manage intubation processes more efficiently and safely.

The CNN structure designed in this study is presented in Table 2. The model uses DL techniques to process and classify image data. There are three Conv2D (convolutional) layers at the beginning of the model. These three layers learn the key features in the images using 32, 64, and 128 filters, respectively. The convolutional layers operate by scrolling over the image, with each filter creating a different feature map. This process helps recognize edges, shapes, and patterns in the image. After the convolutional layers are the MaxPooling2D (pooling) layers, which select the highest value in the images and reduce the size, typically using 2 × 2 windows. Pooling helps the model focus on important features and reduces computational costs. These layers are applied after each convolutional layer, allowing for more intensive learning of features each time.

The next layer is the flatten layer, which converts the 3D feature maps into a single vector. This process is necessary to process the learned features with the fully connected layers. After the flatten layer is the dense layer, which contains 128 neurons and is used to perform classification of the learned features. A dropout layer is also added. Dropout increases the generalization capacity of the model by randomly turning off certain neurons to prevent the model from overlearning. Finally, the output layer of the model contains the dense layer, which performs classification (easy, medium, difficult) using the softmax activation function. Softmax calculates the probability values for each class and predicts the class with the highest probability. The categorical cross-entropy loss function and the Adam optimization algorithm are used to train the model. Using this structure, the model extracts important features from visual data and can accurately predict whether it belongs to a particular class. The CNN model is particularly effective for visual classification tasks and is well-suited for medical image classification applications, such as intubation.

The two deep network models used in the study are EfficientNet and CNN. EfficientNet first extracts basic features using Conv2D layers, then normalizes activations and makes them positive with BatchNormalization and ReLU activation. MaxPooling2D layers preserve important information while reducing model size. For deeper features, Conv2D layers are used again. Next, the flatten layer converts the 3D features into a 1D vector. Dense layers abstract the learned features, and dropout prevents overlearning. Finally, the dense output layer makes predictions of the three classes. This network structure model is presented in Table 3.

## 3. Results

### Results Analysis

In Table 4, the CNN model used to classify the intubation images into easy, medium, and difficult was tested with different batch size and learning rate values to evaluate performance. In general, the accuracy obtained ranged from 86.5% to 87.2%, indicating that the model performed consistently and efficiently under different parameter settings. For a batch size of 32, the highest accuracy of 87.13% was obtained at a learning rate of 0.1. In the same combination, the F1-score also reached the highest value of 87.21%. This shows that a low batch size and a high learning rate can have positive effects on model performance. However, similarly high results were obtained at learning rates of 0.01 and 0.001, which shows that the model works consistently in these ranges. The results for a batch size of 64 were quite similar. In particular, at a learning rate of 0.01, the model achieved a sensitivity of 86.90% and a precision of 87.12%. This combination was also successful in terms of the F1-score (86.98%). With a batch size of 128 and a learning rate of 0.001, the model achieved an accuracy of 87.02%, which is one of the highest accuracy rates reported in the table. The F1-score for this combination was also high, reaching 86.99%. This shows that a high batch size and a low learning rate can also be a powerful combination. In conclusion, the CNN model performed well overall, with the best results generally obtained using batch sizes of 32 and 128 and learning rates ranging from 0.1 to 0.001.

**Figure 3:**
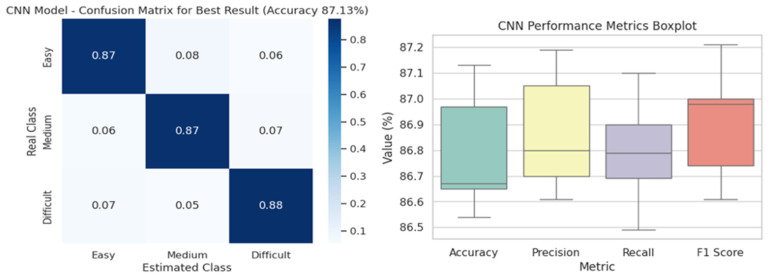
CNN model confusion matrix and distribution of performance metrics across different training configurations.

The confusion matrix in Figure 3 visualizes the classification performance of the model, trained using the best hyperparameter combination of CNN architecture (a batch size of 32 and a learning rate of 0.1) in three classes (easy, medium, difficult). The box plot in Figure 2 shows the overall distribution of the four performance metrics (learning rate, accuracy, precision, sensitivity, F1-scores) of the CNN model. From this plot, the central tendencies, variances, and outliers of the metrics can be easily observed. In particular, the accuracy and precision scores indicate that the model generally performed consistently well overall. Confusion matrix and performance distribution of the CNN model. The confusion matrix illustrates the classification performance obtained using the best hyperparameter configuration (batch size = 32, learning rate = 0.1). The box plot shows the distribution of performance metrics obtained under different training configurations. Each colored box represents a different evaluation metric: accuracy, precision, recall (sensitivity), and F1-score, summarizing their variability across experiments with different batch sizes and learning rates.

In Table 5, key performance metrics, namely, accuracy, precision, sensitivity and F1-scores are presented in detail for the classification processes performed using EfficientNet architecture. The table presents a systematic evaluation of the impact of different batch size (32, 64, and 128) and learning rate (0.1, 0.01, and 0.001) combinations on the model. The model performed best with an accuracy of 88.64% and an F1-score of 87.28%, obtained with a learning rate of 0.01 and a batch size of 32. In this setup, the precision (recall) value was also 88.01%, indicating that the model correctly recognizes the classes. When the learning rate was 0.001, the accuracy decreased (86.30%), while the precision (88.82%) and recall (88.40%) values increased. This means that although the number of correctly classified instances by the model decreased, most of these classifications remained correct. Experiments with a batch size of 64 yielded more stable results. In particular, an accuracy of 87.12% and an F1-score of 87.64% were achieved with a learning rate of 0.001. This result shows that the model achieves stable classification performance and has less instability between classes. In the evaluations with a batch size of 128 and a learning rate of 0.01, although the accuracy values were slightly lower in general, a very high F1-score of 88.53% was obtained. This configuration is considered a preferable option, especially in applications in which a balance between classes is important.

**Figure 4:**
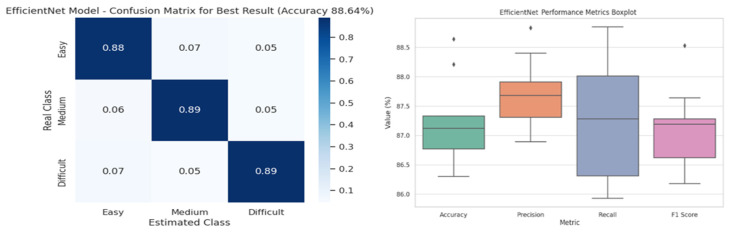
EfficientNet model confusion matrix and distribution of performance metrics across different training configurations.

The confusion matrix in Figure 4 visualizes the model’s classification performance, trained using the best hyperparameter combination in EfficientNet architecture (a batch size of 32 and a learning rate of 0.01) in three classes. The box plot in Figure 3 depicts the overall distribution of the four performance metrics of the EfficientNet model. This visualization helps easily identify the central tendencies, variances, and outliers of the metrics. The F1-score and precision values, in particular, reveal that the model performed consistently well overall. Confusion matrix and performance distribution of the EfficientNet model. The confusion matrix corresponds to the best-performing configuration (batch size = 32, learning rate = 0.01). The box plot visualizes the distribution of evaluation metrics across different training configurations. Each colored box corresponds to one performance metric (accuracy, precision, recall, and F1-score), illustrating the variability of model performance under different parameter settings.

## 4. Discussion

In contrast to the low sensitivity of traditional bedside tests, our EfficientNet-based model classified three different difficulty levels with high accuracy, laying a promising foundation for clinical decision support systems. Difficult or failed endotracheal intubation is one of the most important causes of morbidity and mortality related to anesthesia [27,28]. According to the German Society of Anesthesiology, intubation difficulty occurs in 5–27% of cases, and guidelines have been established to address this difficulty [29]. It has been reported that the most important reason for unsuccessful or inadequate airway management is poor and inadequate assessments [27]. Complications that may occur can be minimized by making the necessary preparations prior to induction in patients with anticipated difficult airways and intubation [30]. One of the reasons for this difficulty is the lack of a uniform index for the risk assessment of intubation difficulties [7]. Algorithms for the management of unexpected difficult or failed tracheal intubation, as well as devices such as gum elastic plugs, video laryngoscopes, and fiberoptic bronchoscopes, are widely used [31]. The ability to predict difficult laryngoscopy allows anesthesiologists to take measures to reduce risk [32]. Although the upper lip bite test has been recommended in recent guidelines as an alternative to the classically used Mallampati and thyromental distance, sternomental distance, and mouth opening measurements, some studies have shown conflicting results [33] in the predictive value of the main signs in preoperative evaluation [34]. In their study, Altinsoy et al. created a different perspective on this issue and found that the skin–epiglottis distance measured by ultrasound is effective in predicting difficult intubation in obese patients [35]. We planned to conduct an innovative study on this issue. Unsuccessful airway management is the major cause of anesthesia-related morbidity (hypoxic brain injury, aspiration pneumonia resulting from pulmonary aspiration, and oral and dental injuries) and mortality [29]. An unpredictable, difficult airway can lead to significant complications and up to 30% of anesthesia-related deaths [36]. In recent years, AI technology has advanced, and image analysis systems have continued to improve. Among them, analytical methods based on CNN have gained prominence [37,38]. By analyzing large volumes of medical data, AI can help clinicians make informed decisions, ultimately leading to improved patient outcomes and efficient resource allocation. Artificial intelligence has the potential to revolutionize healthcare by providing precise diagnoses, personalized treatment strategies, and optimized clinical workflows [39,40]. Regarding AI-based bedside algorithms, devices will soon be developed to assist physicians in assessing the airway during the preoperative anesthesia consultation phase. The data collected during this evaluation can also potentially be used in the preparation of robotic intubation systems, such as the Kepler Intubation System [41].

Our model, based on EfficientNet architecture, outperformed Hayasaka et al.’s CNN-based model. It achieved higher accuracy and F1-scores across key evaluation metrics. In particular, the combination of a batch size of 32 and a learning rate of 0.01 was the most successful configuration, with an accuracy of 88.64% and an F1-score of 87.28%. The precision and accuracy values of our model show that it achieved balanced classification performance and effectively distinguished between classes. Model stability was tested using different hyperparameter combinations, and consistent results were obtained in various scenarios. In this context, the developed model is considered promising in terms of its applicability in clinical settings [42].

Connor et al. classified different cases of difficult intubation using computerized facial analysis. They aimed to categorize intubation into two classes by employing a logistic regression-based model via numerical analysis of facial proportions. They obtained successful results with 90% sensitivity, 85% specificity, and an area under the curve (AUC) of 0.899. However, their study was conducted with a limited number of male patients (*n* = 80), and facial morphology was analyzed by reducing it to a 50-dimensional eigenspace [43]. In contrast, EfficientNet architecture, which was used in our study, performs automatic feature extraction directly from face images and performs three-class (easy, medium, and difficult) classification. It should also be noted that difficult intubation cases are relatively rare in clinical datasets, which may introduce potential class imbalance in machine learning models. In this study, data augmentation techniques were applied during preprocessing to increase the diversity of training samples and improve model robustness. Although the current study focused on a three-class classification framework (easy, medium, and difficult), future studies may also explore binary classification approaches by grouping medium and difficult cases into a single “potential difficult airway” category. Such an approach may further improve the applicability of AI-based systems as clinical screening tools. With an accuracy of 88.64%, a precision of 88.01%, and an F1-score of 87.28% for the best hyperparameter combination, the DL-based approach performed quite well despite distinguishing between more classes. While Connor et al.’s study was performed on a limited sample using traditional statistical methods, the EfficientNet model was successful on more complex classification tasks by using modern AI techniques. This finding suggests that EfficientNet offers a more scalable and powerful solution for clinical decision support systems by enabling a more in-depth structural analysis of facial images.

Kim et al. directly predicted laryngoscopy difficulty using EfficientNet-B5 architecture and a limited number of face and neck images obtained with a smartphone [44]. By integrating information specific to visual angles (frontal, lateral, neck extension, etc.) into the model using a multitask learning approach, the study achieved strong results, such as AUCs ranging from 0.81 to 0.88 and F1-scores ranging from 0.72 to 0.81. Gradient-weighted class activation mapping visually demonstrated that the neck and jaw regions play an important role in classification decisions. In our study, we performed a three-class classification (easy, medium, and difficult) using EfficientNet architecture and achieved very high success rates, such as an accuracy of 88.64%, a precision of 88.01%, and an F1-score of 87.28% in the most successful configuration. While Kim et al. based their model on two-class prediction, we took on a more difficult task by using three-class discrimination and achieved similar or higher performance. We also analyzed in detail the effects of different combinations of batch sizes and learning rates in our study, which provides a more comprehensive assessment of our model’s hyperparameter sensitivity.

The study by Cuendet et al. proposed a fully automated system based on facial morphology to predict difficult intubation [45]. In their study, statistical facial models, which were developed through photographs and videos obtained from 970 patients, enabled parametric extraction of morphologic features, while the random forest algorithm was used in the classification process. Successful results were obtained, with an AUC of 81% in the binary classification scenario and 77.9% for the entire dataset. However, the model was trained on two classes (easy and difficult intubation) only and offered limited explainability. In contrast, our study provided a finer categorization by targeting multiclass intubation discrimination. The model, developed using EfficientNet architecture, was trained on face and neck images in different poses and achieved high performance values, such as an accuracy of 88.64%, a precision of 88.01%, and an F1-score of 87.28%. Furthermore, the stability of the model was analyzed in detail by testing it under different learning rates and batch size parameters. In conclusion, while the work of Cuendet et al. provides an important basis for automation and clinical applicability, our work makes an advanced contribution to predicting difficult intubation with both a more detailed classification structure and higher accuracy rates. Our study aims not only to detect difficult intubation but also to make the intubation process safer and more predictable by providing detailed information for clinical decision support systems.

Tavolara et al. proposed an improved DL approach to predict difficult intubation using frontal facial images only [46]. In their study, a CNN-based ensemble model trained with a large database was used, and classification was performed using multiple sample learning on patient images. In tests on 152 patient data, the model achieved an AUC of 71.05%, well above that of conventional bedside tests, but with a significant trade-off between sensitivity and specificity (e.g., only 44.74% specificity for 90.79% sensitivity). In contrast, our study, which used EfficientNet architecture, obtained stable and high performance values, such as an accuracy of 88.64% and an F1-score of 87.28% in the three-class classification, providing a more stable structure in terms of both overall accuracy and class discrimination. Different hyperparameter configurations were also systematically analyzed to demonstrate the consistency of the model and its performance in class discrimination. In this context, our study fills an important gap in the literature by contributing not only to the detection of intubation difficulty but also to the grading of this difficulty.

Yan and Wei et al. evaluated the Cormack–Lehane classification using only a multilayer perceptron network-based medical decision support system They developed a database to train and test the system using 13 features of 824 patients and reported a classification accuracy of 91.9% [20]. Yan et al. also created a medical decision support system based on support vector machines (SVMs) using 13 physical features for the prediction of tracheal intubation before anesthesia, and examined 264 patients. Their study showed that an SVM-based decision support system can improve the average classification accuracy to 90.53%. This result suggests that the model has great potential for application in clinically assisted diagnosis, with full consideration of the multiple features of airway physical examination [21]. Lazouni et al. reported a Cormack–Lehane classification success rate of 97.26% with the SVM algorithm using measurements and demographic data obtained from patients’ preoperative evaluations [22]. In the study by Celik et al., the demographic variables, clinical tests, and anthropometric measurements of 19 features and 341 patients were evaluated, with the data analyzed in 8 AI algorithms using the WEKA program [47]. Difficult intubation was predicted with 92.85% sensitivity, 96.94% specificity, 93.69% positive predictive value, and 96.52% negative predictive value using this method. Zhou et al. used multiple machine learning ML and DL algorithms to identify difficult airways in patients with thyroid disorders scheduled for thyroid surgery [48]. In addition to these studies, the work of Obuchowicz et al. regarding the use of artificial intelligence has drawn attention to some issues. They stated that in the foreseeable future, artificial intelligence will support physicians rather than replace them, involving the automation of well-defined tasks under human supervision, while clinical integration, physical examination, procedural performance, ethical judgment, and accountability will remain dependent on the physician [49].

Among the algorithms, gradient boosting achieved 100% precision and an AUC of more than 0.8. The model also included parameters such as age, gender, weight, height, and body mass index to improve the ML algorithm. In comparison, our method uses fewer photos and works with general-purpose smartphone cameras, making it easy to apply in a variety of clinical settings without the need for specialized equipment. To provide a clearer and more structured comparison with previously published studies, the main characteristics of several related works in the literature are summarized in Table 6. The comparison includes information about the datasets used, the implemented artificial intelligence models, the classification approaches, and the reported performance metrics. This structured comparison allows the proposed approach to be evaluated in relation to existing studies on the AI-based prediction of difficult tracheal intubation.

As shown in Table 6, most previous studies focused on binary classification of intubation difficulty. In contrast, the proposed study performed a three-class classification using deep learning models (CNN and EfficientNet) based on multi-angle photographic data, providing a more detailed evaluation of airway difficulty. In addition to classification performance, the computational complexity of the proposed models is an important factor for potential clinical implementation. The CNN architecture used in this study consists of three convolutional layers followed by pooling, flatten, and fully connected layers, which provides relatively low computational complexity while maintaining effective feature extraction capability. EfficientNet, on the other hand, is designed using a compound scaling strategy that simultaneously balances network depth, width, and input resolution. This design allows EfficientNet to achieve high classification accuracy while maintaining computational efficiency compared to many conventional deep learning architectures. When compared with previously reported approaches in the literature, such as logistic regression models based on facial morphology (Connor et al.) or random forest models using facial measurements (Cuendet et al.), the proposed deep learning models require higher computational resources during training but provide significantly improved feature representation capabilities. However, once trained, the inference stage of CNN and EfficientNet models can be executed efficiently, making them suitable for real-time clinical decision support systems.

## 5. Conclusions

In our study, instead of using the Cormack–Lehane classification, which is commonly applied in other studies, we categorized intubation as follows. Intubations that were performed easily by a single clinician were classified as easy. Intubations that resulted from external tracheal interventions or required more than one attempt were classified as medium. Finally, intubations that required more than three attempts, involved the use of additional devices, or could not be performed were classified as difficult. This categorization aimed to support the prediction of patients beyond those in the easy group and to enable precautions to be taken before the procedure. We also believe that in a further step, bedside photographs obtained using a smartphone-based application may assist clinicians in predicting difficult intubation. Such an approach may contribute to the development of an objective preoperative assessment tool that can support anesthesiologists in identifying potential airway difficulties before anesthesia induction. However, this study has several limitations that should be considered. First, the dataset used in this study consisted of a relatively limited number of patients, which may restrict the generalization capability of the developed models. Second, the data were collected from a single medical center, and therefore the dataset may not fully represent different patient populations or clinical conditions. In addition, the distribution of intubation difficulty classes may not be perfectly balanced, which could potentially influence the training process of the deep learning models. Although data augmentation techniques were applied to improve model robustness, larger and more balanced datasets would allow for a more comprehensive evaluation of the proposed approach. Furthermore, the study did not include patients from different nationalities, which may limit the generalizability of the results across diverse populations. Future studies should therefore include larger multicenter datasets and more diverse patient groups in order to further validate the proposed method and evaluate its potential clinical applicability in real-world settings. The present study has several limitations that should be considered when interpreting the results. First, the dataset used in this study consisted of a relatively limited number of patients, which may restrict the generalization capability of the developed models. Second, the data were collected from a single medical center, and therefore the dataset may not fully represent different patient populations or clinical conditions. Another limitation is that the distribution of intubation difficulty classes may not be perfectly balanced, which can potentially influence the training process of deep learning models. Although data augmentation techniques were applied to improve model robustness, further studies using larger and more balanced datasets would provide more reliable evaluations. Additionally, the proposed approach relies on photographic data captured under controlled conditions. Variations in lighting conditions, camera positions, and image quality in real clinical environments may affect model performance. Therefore, future studies should evaluate the robustness of the proposed system under different imaging conditions. Finally, although the proposed deep learning models demonstrated promising classification performance, external validation using multicenter datasets and prospective clinical studies will be necessary to confirm the clinical applicability of the proposed method.

## Figures and Tables

**Table 1:** Distribution of patients and corresponding images across the intubation difficulty classes.

Intubation Class	Number of Patients	Number of Images
Easy	38	608
Medium	35	560
Difficult	36	576

**Table 2:** CNN structure designed.

Layer	Type	Description	Output Size
1. Conv2D	Convolutional	The first convolutional layer uses a 3 × 3 kernel with 32 filters.	(148, 148, 32)
2. MaxPooling2D	Pooling	It reduces the image size by applying 2 × 2 pooling, keeping the important features.	(74, 74, 32)
3. Conv2D	Convolutional	The second convolutional layer uses 3 × 3 kernel with 64 filters. It learns more complex features.	(72, 72, 64)
4. MaxPooling2D	Pooling	It reduces the image size again by using 2 × 2 pooling.	(36, 36, 64)
5. Conv2D	Convolutional	The third convolutional layer uses 3 × 3 kernel with 128 filters. It learns deep features.	(34, 34, 128)
6. MaxPooling2D	Pooling	It reduces the image size by using 2 × 2 pooling.	(17, 17, 128)
7. Flatten	Flattening	It converts 3D feature maps into a single vector and prepares it for fully connected layers.	(34,816)
8. Dense	Fully-Connected	The fully connected layer with 128 neurons performs classification using the learned features.	(128)
9. Dropout	Regularization	It applies 50% dropout rate to prevent over-learning.	(128)
10. Dense	Output Layer	It classifies the results using softmax activation for 3 classes.	(3)

**Table 3:** Network structure model (EfficientNet) [26].

Layer	Type	Description	Output Size
Conv2D	Convolutional	32 filters learn basic features with 3 × 3 kernel.	(148, 148, 32)
BatchNormalization	Normalization	Normalizes activations, provides faster learning.	(148, 148, 32)
ReLU Activation	Activation	Activation function that passes positive values by zeroing negative values.	(148, 148, 32)
MaxPooling2D	Pooling	Reduces dimension and keeps important features.	(74, 74, 32)
Conv2D	Convolutional	64 filters learn deeper features.	(72, 72, 64)
MaxPooling2D	Pooling	Dimension reduction process.	(36, 36, 64)
Conv2D	Convolutional	128 filters learn complex features.	(34, 34, 128)
MaxPooling2D	Pooling	Dimension reduction process.	(17, 17, 128)
Flatten	Smoothing	Converts 3D feature maps to 1D vector.	(34,816)
Dense	Fully-Connected	Abstract features are learned with 128 neurons.	(128)
Dropout	Regularization	Prevents over-learning, 50% dropout.	(128)
Dense	Output Layer	Classifies with softmax for 3 classes.	(3)

**Table 4:** Results from CNN.

Model	Batch-Size	Learning Rate	Accuracy %	Precision %	Sensitivity %	F1-Scores %
CNN	32	0.1	87.13	86.61	87.19	87.21
		0.01	86.67	86.69	86.72	87.13
		0.001	86.66	87.05	86.70	86.74
	64	0.1	86.63	86.83	86.61	87.00
		0.01	86.54	86.90	87.12	86.98
		0.001	86.65	86.72	86.98	86.61
	128	0.1	86.97	86.49	86.80	86.72
		0.01	86.86	87.10	86.64	86.98
		0.001	87.02	86.79	87.05	86.99

**Table 5:** Results from EfficientNet.

Model	Batch-Size	Learning Rate	Accuracy %	Precision %	Sensitivity %	F1-Scores %
EfficientNet	32	0.1	88.21	87.57	85.93	86.93
		0.01	88.64	87.15	88.01	87.28
		0.001	86.30	88.40	88.82	87.23
	64	0.1	87.14	86.89	88.85	86.24
		0.01	86.77	88.83	87.53	87.19
		0.001	87.12	87.68	87.28	87.64
	128	0.1	86.43	87.91	86.07	86.18
		0.01	87.02	87.31	86.31	88.53
		0.001	87.33	87.87	86.65	86.62

**Table 6:** Comparison of previous studies on AI-based prediction of difficult tracheal intubation.

Study	Dataset	Method/Model	Classification Type	Performance
Connor et al. [42]	80 patients (facial morphology analysis)	Logistic regression	Binary	Sensitivity 90%, Specificity 85%, AUC 0.899
Cuendet et al. [44]	970 patients (facial images and videos)	Random Forest	Binary	AUC 81%
Tavolara et al. [45]	152 facial images	CNN ensemble	Binary	AUC 71.05%
Kim et al. [43]	Smartphone face and neck images	EfficientNet	Binary	AUC 0.81–0.88
Proposed Study	109 patients (16 images per patient)	CNN + EfficientNet	Three-class (easy, medium, difficult)	Accuracy 88.64%, F1-score 87.28%

## Data Availability

Primary data is available upon reasonable request addressed to the corresponding author.
